# TOOTHBRUSH AND TOWEL HANDLING AND THEIR MICROBIAL QUALITY: THE CASE OF STUDENTS OF UNIVERSITY FOR DEVELOPMENT STUDIES, NYANKPALA CAMPUS, GHANA

**DOI:** 10.21010/ajid.v15i1.5

**Published:** 2020-12-14

**Authors:** Hannah Twumwaa, Betty Asumang, Zarouk Abubakari Imoro, Stephen Wilson Kpordze

**Affiliations:** 1 Department of Ecotourism and Environmental Management, Faculty of Natural Resources and Environment, University for Development Studies, P. O. Box TL 1882, Tamale, Ghana; 2 Department of Biotechnology, Faculty of Agriculture, University for Development Studies P. O. Box TL 1882, Tamale, Ghana; 3 Department of Water, Waste and Environmental Engineering, School of Engineering, University for Development Studies P. O. Box TL 1882, Tamale, Ghana

**Keywords:** Toothbrush, Towel, *Staphylococcus aureus*, *Escherichia coli*, methicillin-resistant

## Abstract

**Background:**

Good toothbrush and towel handling are important considerations in personal hygiene. Thus, this study sought to assess how students of the University for Development Studies handle their toothbrushes and towels and the consequence of that with regards to the microbial quality of these personnel hygiene materials.

**Materials and Methods:**

A total of 100 swap samples were collected (50 toothbrushes and 50 towels) for microbial analysis. Questionnaires were administered to students from whom samples were collected to ascertain information on how they handle toothbrushes and towels. MacConkey agar and Mannitol Salt agar were used to isolate *E. coli* and *S. aureus* respectively, and cefoxitin used to identify the methicillin-resistant *S. aureus* strains.

**Results:**

*E. coli* was present in all sampled towels, while 98% of the sampled toothbrushes contained *E. coli*. It was found that 2% of the respondents kept their toothbrushes in bathhouses, 44% kept them unenclosed in rooms and 54% kept them enclosed in rooms (54%). Also, 48% of the respondents washed their towels once a week, 24% washed once every two weeks, 20% once every month and 8% once a trimester. Moreover, 52% dried their towels in rooms while 48% dried them outside rooms. The occurrence of *S. aureus* was 96% and 94% respectively for the towels and toothbrushes. Of the *S. aureus* isolated, 33.3% of sampled towels and 12.8% of the toothbrushes contained methicillin-resistant *S. aureus*.

**Conclusion:**

This study found that, students are at risk of contracting infectious disease if their personal hygiene behaviours do not changed.

## Introduction

The utmost common hygiene aids used to improve oral health and body-care are toothbrushes and towels respectively. Meanwhile, the human body is teeming with microbial life of which some are pathogenic, others non-pathogenic and another group, those with no apparent functions (Marthakis, 2012). Dirty clothes, including towels, have the potential of harboring microbes and wearing or using them can cause skin infections (Bloomfield *et al.*, 2013).

Hygiene aids like towels and toothbrushes are capable of supporting microbial growth even though these materials are not an ideal environment for their growth (Downes *et al.*, 2008).

In the majority of developing countries, about 80% of illnesses are related to poor home and personal hygiene (Tambekar et al., 2009). Through this, students may get infected with infectious bacteria which can make them ill and be absent from school, thus affecting their academic productivity in school (Rasberry *et al.*, 2017).

In order to increase confidence and self-esteem which is considered as healthy life, one ought to maintain a high level of personal hygiene (Abdul and Hassan, 2012). Students of the University for Development Studies, Nyankpala Campus by observation, simply rinse their toothbrushes with water after use and do not apply any form of sterilization to get rid of microbes. It has also been observed that these students do not maintain a routine in the drying of their towels in the sun. Moreover, due to the irregular supply of water to the campus, students do not often wash their towels.

Research by Dhifaf (2011) and Destiny (2017) revealed that the damp surroundings of the average bathroom permits microbial growth on toothbrushes and on towels. Bathing towels which are constantly kept in areas of high humidity do not only promote bacterial increase but encourages the increase of specific bacteria including fecal coliform bacteria (Destiny, 2017). Also, toothbrushes can become contaminated through contact with the environment, and bacterial survival is affected by toothbrush storage containers (Frazelle and Munro, 2012).

Consequently, this study sought to find out how students handle their towels and toothbrushes and as well, investigate the microbial quality of these towels and toothbrushes.

## Materials and Methods

### Study Area

This research was carried out on the Nyankpala campus of the University for Development Studies (UDS).

The Nyanpkala campus of UDS is 20 km South-West of Tamale metropolis and located in the Tolon District of the Northern Region of Ghana (UDS ICT Directorate, 2011). The campus lies between the latitude 9.25’N and longitude -0.58’W. The campus provides accommodation to students in its three university halls (Union hall, Jeddah hall and Nyankpala hall). There are also private hostels to provide accommodation to students who can afford them.

### Sample Collection

Prior to the starting of this study and sample collection, students` consents were sought. A total of 100 samples (50 towels and 50 toothbrushes) were collected from students for the study. Questionnaire was administered to students whose towels and toothbrushes were sampled for microbial analysis. This was done to collect information on their towel and toothbrush handling practices. Samples were collected between March and May in 2019. Samples were taken from 25 students in the school hostels (Union hall, Jeddah, and Nyankpala hall) and 25 students from private hostels (Esafeed, Richmond and Emmanuel). Students were sampled using the accidental sampling technique.

Surface swabbing of towels were done by rubbing with a sterile cotton swab moistened with sterile physiological saline solution and collected samples kept back in their containers. Toothbrushes of students were collected in sterile bags. Students whose toothbrushes were sampled were provided with new ones. Collected samples were kept in an ice chest containing ice packs and transported to the Spanish Laboratory Complex of the University for Development Studies (UDS), Nyankpala campus for analysis.

### Sample Preparation, Inoculation and Incubation

Bristles and heads from each of the sampled toothbrushes were aseptically removed using sterile scalpel and kept in test tubes containing 9 ml of tryptone soya broth. Tubes were covered and shaken for five (5) minutes. Also, the swab samples from the sampled towels were removed from their containers and kept in labeled test tubes containing 9 ml of tryptone soya broth while constantly swirling for five (5) minutes. Tenfold serial dilutions were performed at four levels for each processed sample. A 0.1 ml each of 10^0^, 10^-1^, 10^-2^, 10^-3^ and 10^-4^ dilutions were taken aseptically and inoculated on prepared Mannitol Salt agar (MSA) and MacConkey agar plates for the isolation of *S. aureus* and *E. coli* respectively. The inoculated plates were incubated for 24 hours at the temperatures of 37 °C for MSA and 44.5 °C for MacConkey agar respectively.

### Biochemical Confirmation of *E. coli* and *S. aureus*

Confirmatory tests were performed for all suspected *E. coli* and *S. aureus* isolates using gram staining, catalase, citrate utilization, indole and urease tests (Maj *et a*l., 2015).

### Methicillin Resistant *Staphylococcus aureus* (MRSA) Determination

A 24-hour culture of all positive *S. aureus* isolates on Nutrient agar were obtained and by employing the Kirby Bauer disk diffusion method, an antibiogram was performed. Bacterial suspensions in test tubes containing 2 ml of 0.89% saline solution were created for all the isolates with their inoculum’s turbidity adjusted to the turbidity equivalent of a 0.5 McFarland standard (which corresponds to approximately 1.5 x 10^8^ CFU/ml). Using sterile cotton swab, the inoculums were carefully swabbed onto prepared Mueller Hinton agar plates in order to achieve homogenous growth coverage of the plates. Swabbed plates were left for about 1-2 minutes before Cefoxitin (FOX 30µg) disks were placed on agar surface with sterile forceps. Impregnated plates were then incubated at 37 ºC for 24 hours. After incubation, zone of inhibition diameter (mm) was measured and recorded. Results were interpreted using the EUCAST 2019 breakpoint guidelines and isolates exhibiting resistance were considered MRSA.

## Results and Discussion

### Toothbrush Handling Practices

Results obtained in this study showed that, 2% of the respondents (representing one male) kept their toothbrushes in bathhouses, 44% (7 females and 15 males) kept toothbrushes exposed to the room environment and 54% (18 females and 9 males) kept toothbrushes enclosed in toothbrush caps and then in lockers. Results on respondents’ knowledge of the ideal place toothbrushes should be kept showed that, 14% (5 females and 2 males) of the students felt it is safest to keep toothbrushes in the bathhouse, 40% (10 females and 10 males) indicated that, keeping toothbrushes exposed in the room was the best microbial-safety practice whiles 46% (10 females and 13 males) reported that, toothbrushes are most secured from microbes when kept in enclosed containers in the room.

Comparing the results of respondents’ knowledge of where toothbrushes should be kept and where the respondents actually kept their toothbrushes, a disparity was realized. The percentage of respondents who recommended the keeping of toothbrushes in bathhouses (14%) were more than those who actually kept their brushes in the bathhouse (2%). This observation could be attributed to the fact that 49% of the respondents shared bathhouses with other students and therefore creating an inappropriate situation for the keeping of toothbrushes in the bathhouse though they wished to. Also, not all the students (46%) who responded that toothbrushes should be kept enclosed actually kept their toothbrushes enclosed. The reason they gave was that toothbrushes in the market did not come with cups. In assessing whether the students were aware that the choice of toothbrush storage had an effect on the oral aid’s microbial quality, 70% were aware of this linkage, whereas 30% were not. The associated reason for why over 50% of the respondents were aware of how storage method could affect toothbrush quality was attributed to the general notion that covering the surfaces of items that enter the mouth protects the mouth from contamination. This general notion is evident in the 46% of the sampled population of this study responding that it was safest to keep toothbrushes enclosed in the room.

Research by Pesevska *et al*. (2016) showed that, it was a common practice for people to keep toothbrushes in bathrooms. This was however in contrast with this study’s finding of having only 2% of the sampled population keeping toothbrushes in the bathhouse. Also, earlier researchers have reported of varying effects of toothbrush storage methods and microbial contaminations. For instance, Mehta *et al*. (2007) found that, storing toothbrushes in caps increased the rate of survival of bacteria. Also Glass (1992) reported that environment with a high humidity increases bacteria survival and also, when there is moisture, bacteria has the ability to survive for more than 24 hours. This means that keeping toothbrushes in the bathhouse would possibly enhance the growth and survival of microbes as compared to keeping the oral aids in the room. The study also found that all respondents did not disinfect their toothbrushes after use and maintained the old practice of simply rinsing toothbrushes in water after brushing. Disturbingly, a study conducted by Sato *et al*. (2005) found that simply rinsing toothbrushes in water led to increased levels of contamination. Thus, this practice should be discouraged.

### Knowledge and the Observance of Recommended Toothbrush Handling Practice

The recommended average lifespan of every toothbrush is three to four months of use (Karibassapa et al., 2011). In this study however, it was found that, only 50% of the respondents adhered to this recommendation whiles 26% were in the habit of changing toothbrushes only when the bristles of the brushes were worn out. On the other hand, the remaining 24% reported that, they changed their toothbrushes when they found it convenient to do so. Interestingly, an assessment of respondent knowledge of the recommended period for toothbrush change revealed the following: 12% of them indicated that toothbrushes should be changed once a month, 14% responded that a change of once every two months was the best practice whiles 56% of them stated that, toothbrushes should be changed every three months and 18% preferred the oral aids be changed only when the toothbrushes no longer served their purpose. Thus, it could be concluded that, half of the respondents observing the change of toothbrushes every three months was a reflection of their knowledge on this issue.

According to Downes *et al*. (2008), even in healthy individuals, toothbrushes get contaminated after the very first use with the level of contamination increasing as the toothbrush is continually used. Interestingly, though 64% of the respondents in this study were aware of this fact they were however, not too keen about keeping their brushes safe from microbial contaminations.

### Towel Handling Practices

Varying towel handling practices were identified in this study. It was found that, 48% of the sampled population dried towels inside their rooms. This comprised of 10 (20%) males and 14 (28%) females. Unanimous reasons given by students (48%) who dried towels inside rooms were: the practice reduced the rate of towels getting dirty as a result of the settling of dusts, another was the prevention of towels from rains during the rainy season. An unexpected reason was the superstitious belief that, ‘evil-forces’ might harm them through towels dried outside rooms.

The remaining 52% of the population dried their towels under direct sunlight. This comprised of 26% each of males and females. It was found that, those (52%) who dried towels outside did so because it was a faster way to get their towels dried. They also indicated that, drying towels outside killed bacteria and prevented bad odor. This is in line with Haider (2018) report that, sun rays (ultra violet rays) kill bacteria and other disease-causing agents and as well, make clothing smell better without perfumes.

### Knowledge on and Observance of Recommended Towel Handling Practice

An assessment of respondent knowledge of how towels should be handled showed that, most of them (90%) appreciated the special care towels required. However, 48% of the students did not handle their towels according to the recommended practice stated by Sturt (2015) and Bradford (2018). According to these authors, towels should be washed every three to four times after they have been used. The study revealed that, 48% of the sampled population washed their towels once every week, 24% once every two weeks, 20% washed towels once every month and 8% of the population washed their towels once a trimester (3- 4 months period). The little adherence of students to the observation of recommended towel handling practice was attributed to carelessness because, 92% of them were aware that, poor towel hygiene including the sharing of towels could lead to microbial infestation. An unfortunate revelation was that, respondents (8%) who did not know microbes could be transmitted through towels indicated that, they did not mind sharing towels with family and friends. The practice of sharing towels is prevalent among the uneducated, thus it was unexpected to find tertiary level students being indifferent about this practice.

### Microbial quality of toothbrushes

Microbial analysis of toothbrushes revealed that, 88% of the toothbrushes belonging to the males had bacteria present on them and 96% of the toothbrushes belonging to the females were contaminated with bacteria. However, the bacteria load on the males’ (4.801×10^2^ CFU for *E. coli* and 3.659×10^2^ CFU for *S. aureus*) toothbrushes were additively more than those on females’ (4.106×10^2^ CFU for *E. coli* and 3.874×10^2^ CFU for *S. aureus*). This study’s findings are in consonance with the findings of Mamai-Homata *et al*. (2016) who reported that, females had the tendency to maintain good oral hygiene than males because of the female’s liking to have a high body image and appearance than the male. However, both male and female toothbrushes sampled contained bacteria of public health concerns. These were *E. coli* and *S. aureus*. There was no statistical significant difference (*E. coli*, p = 0.25 and *S. aureus* p = 0.33) in the bacteria numbers between males and female toothbrushes. The associated reason for the no significant difference in bacteria loads between males and females was that both genders practiced the ‘simple rinsing of toothbrushes after use’ as a cleansing measure. It was also found that, 12.8% of *S. aureus* isolated from toothbrushes were methicillin resistant.

This implied that, careless handling of toothbrushes could lead to cross contamination of bacteria from skin surfaces to toothbrushes.

A comparison between the microbial quality of toothbrushes of students living in the school halls and those living in private hostels showed that all (100%) of the toothbrushes collected from the school halls had microbes present on them whiles 84% of the toothbrushes collected from the private hostels had microbes present on them. This result showed that students in school halls could be more prone to bacterial infections than those in the private hostels. Microbial contamination of toothbrushes could result from bad storage and sterilization methods. A correlation analysis showed no correlation between the length of use of toothbrushes and microbial load. The r value for *E. coli* was 0.06 and that for *S. aureus*, 0.10. This result disagrees with the work of Pesevska *et al*. (2016) who reported of increasing bacteria numbers with increasing length of use of toothbrushes.

### Microbial Quality of Towels

The high bacterial load (Table 1) of sampled towels were attributed to the infrequent washing of towels and the choice of disinfectant used. For example, 46% of the sampled population used only soap and water in washing towels, a method which according to Bloomfield *et al*. (2013) is not effective in killing bacteria.

**Table 1:** Prevalence of microbes on towels and toothbrushes.

	Toothbrushes		Towels	
	*E.coli* (CFU/ml)	*S.aureus* (CFU/ml)	*E.coli* (CFU/ml)	*S.aureus* (CFU/ml)
School Hostels	5.45×10^2^	4.301×10^2^	4.00×10^2^	8.23×10^2^
Private Hostels	3.192×10^2^	3.347×10^2^	9.22×10^1^	9.61×10^2^
Males	4.801×10^2^	3.659×10^2^	1.85×10^2^	6.65×10^2^
Females	4.106×10^2^	3.874×10^2^	2.97×10^2^	1.09×10^3^

The presence of *E. coli* on towels sampled from both hostels was not unexpected because bathrooms of all hostels sampled for this study were close to toilets. Toilets are very likely sources of *E. coli* contaminations. The presence of *E. coli* on towels sampled from both hostels was also attributed to students’ attitudes of failing to wash hands before touching or wiping hands on towels after using the toilets. Though there was no statistically significant difference in *E. coli* load between males and females, their averages showed female towels supported more bacteria numbers than males. This implied that, females had a greater chance of acquiring *E. coli* related sickness/ diseases than males. Females recording higher bacteria numbers could be due to their frequent use of towels. It is well known that, females do more cleansing of body parts with water than males, hence increasing their towel use and the chances of bacterial contamination especially when the source of water is contaminated. This study’s findings agree with Mackenzie (2008) report that females skin teem with more diverse microbes. However, Fischetti (2015) had a contrasting finding where males were found to shed more bacteria from their body into the environment than females.

Out of the total of 50 samples collected from both hostels, *S. aureus* was isolated from 48 samples *.S. aureus* is a normal bacteria flora on the skin, thus its detection on 96% of sampled towels was expected. *S. aureus* is also resistant t o drying (Sattler *et al.*, 2004) and this partly explained why they were as well detected on towels that were indicated to be dried outside. Though there was no statistical significant difference in *S. aureus* load between males and females, their averages showed female towels supported more of bacteria (*S. aureus*) numbers than males. This observation was attributed to the female’s frequent use of towels as already indicated for *E. coli*.

Though *S. aureus* is identified as a normal bacteria flora of the skin surface, the methicillin resistance strains are of concern because of their pathogenicity. The prevalence rate of MRSA among the isolated *S. aureus* were found to be 33.3%. Resistance of *S. aureus* to cefoxitin has previously been reported (Furuno *et al.*, 2008; Manzur *et al.*, 2008). Thus new antibiotics with high potencies need to be developed and drug abuse controlled to contain the spread of MRSA.

## Conclusion

It was found that, most students do not observe recommended toothbrush and towel handling practices though had good knowledge of these standards. Also, this study found that students’ toothbrushes and towels were contaminated with *E. coli* and *S. aureus*. Moreover, some *S. aureus* were found to be *methicillin resistant Staphylococcus aureus* thus posing as a serious health threat to students.

**List of Abbreviations:**
ICT: Information and communications technology
MRSA: Methicillin Resistant *Staphylococcus aureus*
MSA: Mannitol Salt agar
CFU: Colony forming units forming units
